# Spanish Nurses’ Knowledge about Palliative Care. A National Online Survey

**DOI:** 10.3390/ijerph182111227

**Published:** 2021-10-26

**Authors:** Antonio Martínez-Sabater, Pilar Chover-Sierra, Elena Chover-Sierra

**Affiliations:** 1 Nursing Department, University of Valencia, 46010 Valencia, Spain; Antonio.Martinez-Sabater@uv.es (A.M.-S.); pilarchoversierra@gmail.com (P.C.-S.); 2 Nursing Care and Education Research Group (GRIECE), GIUV2019-456, Faculty of Nursing and Podiatrics, University of Valencia, 46010 Valencia, Spain; 3 Grupo Investigación en Cuidados (INCLIVA), Hospital Clinico Universitario de Valencia, 46010 Valencia, Spain; 4 Hospital Clinico Universitario de Valencia, 46010 Valencia, Spain; 5 Internal Medicine Department, Consorcio Hospital General Universitario de Valencia, 46014 Valencia, Spain

**Keywords:** palliative care, nursing, PCQN-SV, knowledge, nurses’ education

## Abstract

(1) Background: Nurses can find people with advanced diseases or in their last days of life during their professional careers and in many different care settings. For this reason, they need to have at least a basic level of palliative care education since they are the professional cohort treating these patients in a very close way. This research aims to determine the level of knowledge in palliative care of Spanish nurses and establish any possible difference based on their experience and training in palliative care. (2) Methods: A cross-sectional design using survey methods (distributed an online questionnaire) aimed at Spanish registered nurses. A validated questionnaire (PCQN-SV) was used to determine the level of knowledge in palliative care; information on some variables to characterize the population was also collected (experience and education in palliative care, years of professional experience, academic level, and others). Uni and bivariate descriptive analyses were performed. A binary logistic regression model was also developed to identify those variables that influenced obtaining results higher than the population’s average. (3) Results: Spanish nurses have a medium–low level of knowledge in palliative care, higher in those who have previous experience or education in this area. Statistically significant differences were also found according to the area in which their caring activity was developed. (4) Conclusions: It is necessary to implement strategies for the basic training of nursing professionals in palliative care to offer quality care to people in advanced stages of illnesses or at the end of their lives.

## 1. Introduction

Palliative care means “improving the quality of life of patients and their families in the face of drawbacks associated with life-threatening diseases, through the prevention and relief of suffering, based on early identification and proper management and treatment of pain other physical, psychosocial, and spiritual problems” [1,2,3].

At first, palliative care emerges in the context of caring for people with oncological diseases, aiming to control their pain and other symptoms. However, nowadays, the inclusion of a palliative care approach is considered in the attention of any person with a chronic and degenerative disease. It is emphasized in early-onset diseases to help patients and families maintain a better quality of life [1,4,5].

The philosophy of palliative care is supported on the idea of maintaining the best quality of life for people affected by degenerative diseases and especially in the advanced stages of the disease [1], mainly since the 1960s, when Cicely Saunders began to be interested in these people’s care and gave rise to the ‘hospice’ movement [6].

The surge in life expectancy and the chronification of pathologies have increased interest in people’s care in the advanced and final stages of their pathological processes [1,7,8,9,10]. In this way, work is being done to develop palliative care plans aimed at specific health problems and train professionals who care for patients suffering from them. [11,12,13,14,15]

Nurses are one of the bases in care for people with diseases in advanced and end-of-life stages, to whom it is necessary to show this consideration. However, to provide quality care, they need to have adequate training in these aspects, especially if professionals can find people who may require this kind of attention in any field of work and not only if working on a specific resource of palliative care [16,17,18]. Nurses’ knowledge contributes to the holistic care of people with chronic and life-threatening diseases and their families. Hence, the possession of adequate information and skills is essential when dealing with death and dying [19].

Research conducted in recent years and several countries among nurses working in different assistance areas has shown that they present knowledge deficits when managing this sort of patient. This lack of skills has been established by results obtained in qualitative studies for their evaluation and qualitative studies that gathered the perceptions of such professionals on their need for more training in palliative care. It must be said that these studies have been carried out among nurses who developed their occupations in different workplaces. Some differences can be found among the diverse levels of care despite these deficits being identified globally [18,20,21,22,23].

For this reason, it is essential for any nurse, as recommended by the various scientific associations and experts in this field, to be taught at least a basic level of palliative care. This knowledge would allow them to work with people susceptible to receiving this type of attention, whatever the area in which they develop their professional activity [20,24,25,26,27].

In recent years, this training has been progressively included in the degree programs of different faculties in Spain, despite not being offered in all of them [28,29]. In addition, professionals can have access to different ongoing training programs on this topic, always depending on their personal and professional interests.

Few studies have been developed in our country to analyze Nurses’ education and knowledge in palliative care. Hence, this study was decided to be carried out nationwide to determine the level of knowledge in palliative care of Spanish nursing professionals and establish possible differences in this level of knowledge according to their experience and training in palliative care.

## 2. Materials and Methods

### 2.1. Design

A descriptive cross-sectional study was carried out.

### 2.2. Participants

A sample of Spanish Registered Nurses was recruited using the Internet and the social networks Facebook and Twitter, as explained in previously published research [30]. This method has shown its usefulness in developing a snowball sampling and recruiting a significant number of participants in a short period. Facebook groups related to palliative care and Twitter hashtags helped to address to study population.

The sample size was calculated considering that about 200,000 nurses are registered in the Spanish National Health System (4.2 per 100,000 inhabitants). The optimal sample size to get a proportion of 40% of nurses with a good level of knowledge in palliative care is 892. Therefore, the final number of participants in the study (1114 RNs) could be considered adequate to our interests.

### 2.3. Data Collection

Data were collected between November 2018 and June 2019 using an online questionnaire developed with Google Formular^®^, which included sociodemographic data of participants and their answers to the PCQN-SV.

#### 2.3.1. Sociodemographic Data

The first part of the questionnaire collected information about the descriptive population’s features that would later make it possible to explain the differences in obtained results. Among other data, it collected information on the age and gender of the participants, years of professional experience, level of training, the area where they developed their professional activity, and whether they had experience or training in palliative care.

#### 2.3.2. PCQN-SV

The instrument used to measure this level of knowledge is the Spanish version of the Palliative Care Quiz for Nurses (PCQN-SV). It is a tool with 20 questions of true/false with queries about philosophy and principles of palliative care (4 questions), psychosocial aspects (3 questions), and symptoms’ control (13 questions), developed at the University of Ottawa.

The PCQN has been used in research developed in different countries, either the original in English or its versions in other different languages [31,32,33,34,35,36,37,38,39,40,41,42,43,44,45,46,47,48,49,50,51]. The authors validated the Spanish version of the questionnaire (PCQN-SV) using a translation and back-translation process. The PCQN-SV has shown adequate content validity and internal consistency indexes in the different studies where it has been applied [30,52,53]. In this study, the results about the questionnaire’s reliability are also very similar to published research, with Cronbach’s alpha of 0.64 and KR-20 of 0.72, respectively.

### 2.4. Ethical Considerations

The first page of the data collection form included an informative letter to explain the study objectives and to inform participants about data confidentiality and anonymity. The ethics committee of the University of Valencia indicates in its normative that, in those studies in which identification data of the participants is not required or in opinion surveys, it is enough to clarify their anonymity. Any personal data or contact information that could help identify the participant was included in the form, so data collection was completely anonymous. Although it could be understood that only those people who wanted to participate in the study would answer the questionnaire, their intention to participate was explicitly stated in a YES/NO question at the end of this informative letter. This question should be answered compulsorily before access to the data collection instrument itself.

### 2.5. Data Analysis

SPSS version 24 was used to analyze the data.

Univariate and bivariate descriptive analyses of the data collected were performed. In the bivariate analysis, non-parametric tests (Mann–Whitney’s U and Kruskal–Wallis test) were used because of the violation of the normal distribution assumption.

A binary logistic regression model was also developed to identify predictors of results (percentage of correct answers in PCQN). In this binary logistic regression model, the independent variable became dichotomous (1 = good knowledge/0 = poor knowledge), defining “poor knowledge” as obtaining a percentage of correct answers superior to 60% of correct answers obtained in the whole questionnaire. Categorical and numerical variables were used as explanatory ones.

## 3. Results

### 3.1. Features of the Studied Population

Finally, 1114 registered nurses from all over Spain participated, especially women, with an average age of almost 39 years and a professional experience of around 15.

The participants’ features are shown in Table 1.

Many participants (43.4%) developed their care activity in acute care hospitals, and 22.5% currently work in primary care. On the other hand, only 52 of them (4.7%) worked in specific resources of palliative care or in-home hospitalization units. Besides this, 101 participants (9.1%) reported working in chronic diseases hospitals or long-term care centers, and only 40 professionals claimed they worked in centers for the attention of the elderly.

Most professionals reported receiving it through ongoing education courses, and 51.5% reported receiving it in the last five years regarding specific palliative-care training.

When analyzing the relationship between professional experience and the type of education in palliative care received, 37.1% of professionals with less than 10 years of professional experience reported having received such content during their university education. However, those with more than 25 years of professional experience received it mostly in postgraduate courses or ongoing education, reaching up to 51.5%.

### 3.2. Nurses’ Level of Knowledge in Palliative Care

The results for each item composing the questionnaire are shown in Table 2 as the percentages of right and wrong answers.

Table 3 shows the percentage of right and wrong answers in the global questionnaire and in each subscale that compose it.

### 3.3. Variables That Determine the Level of Knowledge in Palliative Care of Participant Nurses

Correlations between the years of professional experience (both global and in the field of palliative care), hours in palliative care training reported by participant nurses, and percentages of correct answers (both in the global questionnaire and each of the subscales) were analyzed. The results are shown in Table 4, in which the existence of a direct linear relationship between variables can be observed.

When studying the percentage of right answers in the global questionnaire, it is observed that participants with experience in palliative care obtain higher percentages of correct answers and lower percentages of errors than those who report having no experience in this area. The same trend is also noted in the case of participants who report having training in palliative care. Table 5 shows the percentages of right and wrong answers in the global questionnaire and each subscale, based on the participants’ experience and training in palliative care.

When analyzing the results obtained by the participants based on the health setting where they carry out their nursing activity, differences in percentages of right and wrong answers in the questionnaire were also found. These differences, shown in Table 6, are statistically significant, according to the Kruskal–Wallis test.

### 3.4. Explanatory Model on the Level of Knowledge in Palliative Care of Spanish Nurses

Table 7 presents the odds ratio assigned to each variable included in the logistic binary regression model. Factors influencing the achievement of a percentage higher than 60 correct answers in the overall PCQN-SV could be explained through this model.

Thus, it is seen that nurses who have received training in palliative care are 58.1% more likely to be classified as “having good knowledge in palliative care” than those who have not received it. Nurses with experience in palliative care are 54.5% more likely to obtain a score higher than 60 than those without working experience in this field.

Regarding the years of experience in palliative care, the model indicates that for each year of experience achieved in this area, the probability of being classified as “having good knowledge” increases by 3%.

## 4. Discussion

This research has pointed out that Spanish nurses have a medium–low level of knowledge in palliative care, higher in those who have previous education in this field, both practical and theoretical ones.

These results about the influence of education in palliative care show the need to implement basic training programs in palliative care, including undergraduate training or ongoing education courses aimed at professionals. We should not forget that nursing professionals can find patients susceptible to needing palliative care in any field we develop our healthcare activity, so they need to have at least a basic level of knowledge.

The professionals participating in this study and multiple studies developed in our country and other countries have identified this need for more and better training in palliative care. In various studies, the professionals themselves express their feelings of not being sufficiently trained in managing patients with the need to receive palliative care [35,46,54,55,56,57].

The detailed analysis of the number of right and wrong answers to the items that compose PCQN-SV can serve as the basis for developing future palliative care education programs since it helps to identify misconceptions that could be solved through this training. Besides, the PCQN-SV allows, as other authors have done in their work, to evaluate the improvement of knowledge generated through a training program through intervention studies with a pretest–posttest design [16,34,58,59,60].

Focusing on the precise results obtained by the participants, an average percentage of correct answers in PCQN of 61.4% was found. These results are better than those obtained when piloting the Spanish version of the PCQN [52], a study conducted in Florida among pediatric nurses [61], a study to validate the French version of the PCQN [62], and those obtained in research conducted in countries where palliative care has very little development, such as Ethiopia [40,63], Saudi Arabia [64], Mongolia [65], China, [48] Nepal [51], Jordan [50,66], and Indonesia [45].

On the other hand, these results are similar or slightly lower than those obtained in other studies among nurses who developed their professional activity in various work environments or countries like Korea, Canada, Australia, Ireland, and the USA. In these studies, authors reported that participating nurses obtained results superior to 60% of correct answers [39,47,67,68].

In addition to that, when evaluating these results separately for each questionnaire subscale, it is observable that Spanish nursing professionals obtain better results in the subscale of philosophy and principles of palliative care and the worst in psychosocial aspects subscale. Ronaldson [43] and Raudonis [68], who measured the level of knowledge in palliative care among nurses working in nursing homes, found that the highest percentages of correct answers were obtained in the psychosocial aspects scale (62% and 75.67%, respectively). The worst results were obtained in the philosophy scale (50% and 57.25%, respectively). Nepalese nurses [51] also obtained their lowest scores in the philosophy subscale (34.5% correct answers). Nurses participating in the study conducted in Mongolia [65] obtained their worst scores in psychosocial aspects with 25.5% of correct answers, scores very similar to those obtained by Chinese nurses [69] and nursing students [70]. In the study conducted in Saudi Arabia by Aboshaiqah [50], the scale of psychosocial aspect obtained only a 20.33% of correct answers.

These poor results in the subscale of psychosocial aspects of palliative care indicate the need to reinforce these contents in training programs, focusing on other aspects such as symptomatic management and basic palliative care concepts. This fact is also identified in studies exploring educational needs in palliative care for both students and professionals. Indeed, both groups refer to the need for training in these psychosocial aspects of palliative care [16,23,48,49,71,72,73,74,75].

Regarding the influence of experience in the level of knowledge in palliative care of nursing professionals, it was demonstrated that those participants who reported having experience in this field obtained better results than those who did not. This leverage was also reflected in the relationship between years of experience in palliative care and results obtained in the PCQN-SV. This influence of experience shows the importance of the knowledge acquired in the work environment. Nevertheless, this knowledge needs a theoretical basis that underlies it, as the results suggest when revealing a more significant effect of education than practical experience.

Hence, regarding having received training in palliative care and its influence on the level of knowledge, participants who reported having received training obtained higher percentages of correct answers, both in the global questionnaire and in each of the subscales that composed it. Regarding the number of training hours, it is also remarkable that there is also a relationship among the results obtained in the questionnaire.

Many other studies about Nurses’ knowledge in palliative care have analyzed the percentage of subjects with experience or education in palliative care [39,48,49,68,76]. Although they did not always analyze differentiated results based on these characteristics, they usually show a direct relationship between experience or training in palliative care and better results in palliative care knowledge. Other works have tried to evaluate the role of training in palliative care in the context of some educational plans and to analyze its effects [16,17,42,60,69].

Concerning the model developed to explain the achievement in the questionnaire of a percentage of correct answers of more than 60%, having both training and experience in palliative care could explain these results. These results highlight the importance of theoretical and practical training for nurses in this field to offer their patients the best quality care possible. They also highlight the need for ongoing training and updating in this ambit [29,77,78,79,80].

Training is essential, but this training must be updated. Nurses participating in this study were asked if they had received such training in the last five years, with 67% answering affirmatively and obtaining better scores in PCQN-SV. Furthermore, when relating the years of professional experience and having received PC training, nurses with less professional experience reported receiving PC training during their undergraduate education. These results reflect the progressive implementation of palliative care contents in nursing university studies, as recommended by the different scientific societies regarding the need for any nursing professional to receive basic training in palliative care during their undergraduate training [20,72,77,79,81,82].

One of the main limitations of this study is related to the methodology of the selection of the participants. Even though the final number of these can be considered high, it cannot be maintained that they are a representative sample of Spanish nursing professionals since no random sampling was done. The characteristics of the participants are indeed quite heterogeneous, and nurses from different areas of our country collaborated. However, at no time can it be said that it is a representative sample.

The purpose of this research was to give an overview of the level of knowledge of nursing professionals in the field of palliative care. It is thought that in this study, the situation of Spain was accurately shown in this regard. In addition, this work is part of the adaptation and validation project of the PCQN-SV.

## 5. Conclusions

Spanish nursing professionals have limited knowledge in palliative care, although this knowledge is higher in those who have experience or training in this field.

The analysis of Nurses’ knowledge in palliative care permits identification aspects to be reinforced in a palliative care training program aimed at these professionals.

## Figures and Tables

**Table 1:** Features of the studied population.

	Mean ± SD	Range	*n*	%
Age	38.92 ± 10.76	20–67		
Gender				
Feminine			866	77.7
Masculine			248	22.3
Professional experience (years)	15.05 ± 10.74	0–45		
Less than 10 years			437	39.2
Among 10 and 25 years			425	38.2
More than 25 years			252	22.6
Academic qualification				
Basic nursing degree			677	60.8
Master’s degree			288	25.9
PhD			55	4.9
Nurse specialist			94	8.4
Experience in palliative care				
YES			518	46.5
NO			596	53.5
Experience in palliative care (years)	2.47 ± 4.99	0–43		
Training in palliative care				
YES			711	63.8
NO			403	36.2
Training in palliative care (type)			711	
University education			221	31.1
Ongoing training			313	44
University + ongoing training			55	7.7
Postgraduate courses			62	8.7
Master’s degree			31	4.3
Master’s + ongoing training			15	2.1
Others			14	2
Training in palliative care (hours)	101.10 ± 395.99	0–7200		

SD. Standard deviation.

**Table 2:** Right and wrong answers for each question composing the PCQN-SV and its subscales.

		Right Answers		Wrong Answers	
Subscale	Item	*n*	%	*n*	%
Philosophy and principles	1 Palliative care is appropriate only in situations where there is evidence of a downhill trajectory or deterioration.	934	83.8	168	15.1
	9 The provision of palliative care requires emotional detachment.	832	74.7	225	20.2
	12 The philosophy of palliative care is compatible with that of aggressive treatment.	715	64.2	282	25.3
	17 The accumulation of losses renders burnout inevitable for those who seek work in palliative care.	505	45.3	492	44.2
Psychosocial aspects	5 It is crucial for family members to remain at the bedside until death occurs.	194	17.4	882	79.2
	11 Men generally reconcile their grief more quickly than women.	648	58.2	197	17.7
	19 The loss of a distant or contentious relationship is easier to resolve than the loss of one that is close or intimate.	557	50	471	42.3
Symptoms’ control	2 Morphine is the standard used to compare the analgesic effect of other opioids.	627	56.3	310	27.8
	3 The extent of the disease determines the method of pain treatment.	752	67.5	323	29
	4 Adjuvant therapies are important in managing pain.	1079	96.9	13	1.2
	6 During the last days of life, the drowsiness associated with electrolyte imbalance may decrease the need for sedation.	435	39	521	46.8
	7 Drug addiction is a major problem when morphine is used on a long-term basis for the management of pain.	680	61	370	33.2
	8 Individuals who are taking opioids should also follow a bowel regime.	1056	94.8	24	2.2
	10 During the terminal stages of an illness, drugs that can cause respiratory depression are appropriate for the treatment of severe dyspnea.	654	58.7	313	28.1
	13 The use of placebos is appropriate in the treatment of some types of pain.	701	62.9	310	24.8
	14 In high doses, codeine causes more nausea and vomiting than morphine.	489	43.9	203	18.2
	15 Suffering and physical pain are synonymous.	915	82.1	177	15.9
	16 Demerol is not an effective analgesic in the control of chronic pain.	429	38.5	441	39.6
	18 Manifestations of chronic pain are different from those of acute pain.	930	83.5	140	12.6
	20 The pain threshold is lowered by anxiety or fatigue.	527	47.3	538	48.3

**Table 3:** Percentages of right and wrong answers in global PCQN-SV and its three subscales.

	Mean	CI (95%)	SD
Right answers			
Global	61.38	60.55–62.20	14.08
Philosophy and principles	67.23	65.76–68.71	25.06
Psychosocial aspects	41.89	40.14–43.64	29.85
Symptom control	64.03	63.09–64.98	16.07
Wrong answers			
Global	28.74	27.99–29.49	12.71
Philosophy and principles	26.05	24.67–27.44	29.85
Psychosocial aspects	46.41	44.77–48.05	27.86
Symptom control	25.46	24.63–26.28	14.03

CI Confidence interval; SD. Standard deviation.

**Table 4:** Relationship between results obtained in PCQN-SV, years of professional experience (both global and in palliative care), and hours of training in palliative care of the studied population.

		Professional Experience (years)		Experience in PC (years)		Training in PC (hours)	
		rho	*p*	rho	*p*	rho	*p*
Global	Right answers	0.04	0.17	0.24	<0.001	0.24	<0.001
	Wrong answers	−0.01	0.61	−0.09	<0.01	−0.08	<0.01
Philosophy and principles	Right answers	−0.01	0.66	0.11	<0.001	0.09	<0.001
	Wrong answers	0.02	0.41	−0.04	0.16	−0.02	0.37
Psychosocial aspects	Right answers	0.01	0.94	0.12	<0.001	0.09	<0.01
	Wrong answers	0.01	0.73	−0.03	0.24	−0.04	0.15
Symptom control	Right answers	0.07	<0.05	0.23	<0.001	0.24	<0.001
	Wrong answers	−0.04	0.15	−0.10	<0.001	−0.10	<0.01

PC. Palliative care.

**Table 5:** The percentages of right and wrong answers in the global questionnaire and each subscale.

		Right Answers			Wrong Answers		
		Mean	SD	*p*-Value	Mean	SD	*p*-Value
Experience in PC	Global PCQN						
	Yes	63.8	14.3	*p* < 0.001	28.2	13.2	*p* = 0.16
	No	59.2	13.6		29.2	12.2	
	Philosophy and principles						
	Yes	69.4	25.2	*p* < 0.05	25.6	23.9	*p* = 0.46
	No	65.3	24.8		26.4	23.4	
	Psychosocial aspects						
	Yes	45	28.9	*p* < 0.01	45.8	27	*p* = 0.57
	No	39.2	30.4		46.9	28.6	
	Symptom control						
	Yes	66.4	16.8	*p* < 0.001	24.9	14.9	*p* = 0.06
	No	61.9	15.1		25.9	13.2	
Training in PC	Global PCQN						
	Yes	63	13.9	*p* < 0.001	27.9	12.4	*p* < 0.01
	No	58.5	14		30.2	13.1	
	Philosophy and principles						
	Yes	68	25.3	*p* = 0.11	34.8	24	*p* = 0.52
	No	65.9	24.5		39.1	22.9	
	Psychosocial aspects						
	Si	43.9	29.6	*p* < 0.01	45.3	27.8	*p* < 0.05
	No	38.4	30		48.4	27.8	
	Symptom control						
	Si	60.9	15.9	*p* < 0.001	27.2	14.5	*p* < 0.01
	No	65.8	15.9		24.4	13.6	

SD: Standard deviation; PC. Palliative care.

**Table 6:** Differences in results obtained in PCQN-SV according to the health setting where the participants worked.

	Right Answers			Wrong Answers		
	Mean	SD	*p*-Value	Mean	SD	*p*-Value
Global PCQN						
Acute care hospital	59.9	13.8		29.5	12.7	
Primary care	61.7	15		27.6	12.9	
Long-term care hospital	61	13.7	*p* < 0.001	30	12.2	*p* < 0.01
Nursing home	59.4	13		31	12.9	
Palliative care unit	69.6	15.5		23.8	13.7	
Philosophy and principles						
Acute care hospital	63.6	24.8		28.6	24.2	
Primary care	67.8	25.5		24.6	23.3	
Long-term care hospital	70.3	24.2	*p* < 0.001	24	23.1	*p* < 0.05
Nursing home	66.1	27.5		30.3	26.2	
Palliative care unit	76.3	26.6		21.7	23.4	
Psychosocial aspects						
Acute care hospital	38.4	30		47.4	27.3	
Primary care	41.4	29.5		47.7	28.5	
Long-term care hospital	44.9	28.4	*p* < 0.01	44.9	26.8	*p* = 0.63
Nursing home	41.3	32.8		45.2	31.1	
Palliative care unit	48.2	29.7		42.1	28.7	
Symptoms’ control						
Acute care hospital	63.6	15.6		25.6	13.7	
Primary care	64.5	16.5		23.9	13.7	
Long-term care hospital	61.4	16.8	*p* < 0.01	28.5	15.2	*p* < 0.01
Nursing home	61.5	15.2		28	14	
Palliative care unit	72.5	17.3		20.2	15.5	

SD. Standard deviation.

**Table 7:** Odds ratio and coefficients of variables included in the logistic regression model.

	β	Wald	*p*-Value	OR	OR (CI 95%)
Experience (YES/NO)	0.44	9.95	<0.01	1.545	1.179–2.024
Training (YES/NO)	0.46	12.52	<0.001	1.581	1.227–2.037
Experience (years)	0.03	4.28	<0.05	1.030	1.002–1.060

OR: odds ratio; CI Confidence interval.

## Data Availability

Please refer to suggested Data Availability Statements in the section “MDPI Research Data Policies” at https://www.mdpi.com/ethics. You might choose to exclude this statement if the study did not report any data.
